# Adapting and Co-Producing a Psychological First Aid Intervention for Care Home Staff: A Person-Based Approach to Enhance Workforce Resilience

**DOI:** 10.3390/ijerph23040431

**Published:** 2026-03-30

**Authors:** Mariyana Schoultz, Alexandra Kirton, Jason Scott, Darren Flynn, Michelle Beattie, Sarah Denford, Geoffrey L. Dickens

**Affiliations:** 1 Department of Nursing, Midwifery & Health, Northumbria University, Coach Lane Campus, 15 Coach Lane, Newcastle upon Tyne NE7 7TR, UK; 2 Centre for Rural Health Sciences, University of the Highlands and Islands, Old Perth Rd, Inverness IV2 3JH, UK; 3 Health Protection Research Unit, University of Bristol, Beacon House, Queens Rd, Bristol BS8 1QU, UK

**Keywords:** psychological first aid, care homes, workforce resilience, co-production, qualitative research, person-based approach, trauma-informed care

## Abstract

**Highlights:**

**Public health relevance—How does this work relate to a public health issue?**

- Care home staff experience frequent exposure to traumatic events, increasing risk of psychological distress and burnout.
- Psychological First Aid (PFA) is an evidence-based approach recommended by WHO, but its adaptation for care homes has been underexplored.

**Public health significance—Why is this work of significance to public health?**

- This study co-produced an adapted PFA intervention using a person-based approach, ensuring contextual relevance and acceptability.
- It specifies how standard WHO PFA materials were adapted by adding care-home-specific scenarios, clarifying language, and restructuring the content into short, modular formats compatible with care-home workflows.

**Public health implications—What are the key implications or messages for practitioners, policy makers and/or researchers in public health?**

- The adapted training outlines essential implementation requirements (e.g., protected time, blended delivery, printable materials) and may support more consistent responses to distressing events; however, perceived usefulness should not be interpreted as demonstrated outcomes.
- Findings offer a transferable model for adapting psychological interventions to other high-stress care environments internationally.

**Abstract:**

Care home staff are routinely exposed to stressful and traumatic events, increasing risks of psychological distress, burnout, and reduced workforce resilience. Psychological First Aid (PFA), recommended by the World Health Organization, provides an evidence-based framework for delivering immediate emotional and practical support; however, its adaptation for care home contexts is limited. This study aimed to co-produce and adapt an existing PFA training resource for care home staff using a person-based approach (PBA) to enhance contextual relevance, acceptability, and feasibility. A two-phase qualitative design guided by PBA principles was used. Phase 1 integrated stakeholder workshops, semi-structured interviews, and literature review to generate guiding principles, a logic model, and preliminary training content. We adapted the WHO PFA “Look–Listen–Link” framework alongside existing open-access materials. Phase 2 used think aloud interviews to optimize usability and contextual fit. Thematic and sentiment analysis identified key needs: high exposure to traumatic events, inconsistent organisational support, desire for measurable skill development, the importance of transferable competencies, and motivational factors. Participants emphasized the need for flexibility, inclusivity, and realistic care-home-specific examples. Adaptations included bite-sized interactive modules, blended delivery options, and reflective exercises. The final co-produced intervention aligns with trauma-informed principles and organisational realities. Further work is needed to access feasibility, acceptability, and fidelity in real-world settings, offering a transferable model for adapting psychological interventions in other high-stress care environments internationally.

## 1. Introduction

The mental health and wellbeing of care home workers are critical for providing high-quality care and sustaining residents’ wellbeing [1,2]. Care home staff play a central role in meeting residents’ physical, emotional, and social needs, yet they routinely encounter stressors and resident-related critical incidents such as sudden deaths, deterioration of health without immediate medical support, and restrictions on family visits during infectious disease outbreaks [3,4]. Recent UK data indicate that between 40–60% of care home workers report symptoms consistent with high stress or burnout, and turnover remains substantially higher than in NHS settings [5]. These experiences expose staff to significant emotional strain and trauma, which can negatively affect their mental wellbeing, job satisfaction, and retention.

Evidence highlights that poor mental wellbeing among care home staff contributes to burnout, absenteeism, and workforce instability [6]. Addressing these challenges requires interventions that are both evidence-based and context-sensitive. However, in the absence of care home specific evidence, adapting established, relevant interventions from other healthcare settings is a strategic and resource-efficient approach. These adaptations must be grounded in the realities of the care home environment to ensure acceptability, feasibility, and effectiveness. To avoid conflating concepts, in this study “stressors,” “trauma exposure,” and “workforce resilience” are treated as distinct constructs: stressors refer to challenging daily events; trauma exposure refers to emotionally overwhelming or distressing incidents; and resilience refers to the capacity of staff to maintain functioning despite such experiences.

Psychological First Aid (PFA) is a trauma-informed approach recommended by the World Health Organization (WHO) for providing immediate psychological support and practical assistance to individuals following a traumatic event or critical incident, events that are sudden, unexpected, and outside the range of normal experience, often involving perceived threats to life or emotional loss [7]. PFA aims to promote safety, calm, and self-efficacy while helping individuals access social and practical support and to begin coping and commence recovery. Originally developed for use in humanitarian and disaster settings, PFA has since been applied to diverse contexts, including healthcare, education, and emergency response, including during the COVID-19 pandemic [8,9,10] which intensified stressors for care home workers, revealing a pressing need to prioritize mental health, resilience, and workforce sustainability [11]. Staff shortages, moral distress, and isolation further exacerbated risks to wellbeing. Supporting care home workers’ mental health is therefore essential not only for individual wellbeing but also for continuity and quality of resident care.

Evidence indicates that PFA training can improve coping, reduce anxiety, and mitigate symptoms of post-traumatic stress [12,13,14,15,16]. Training participants often report enhanced confidence and communication skills when providing emotional support to others [17]. However, training formats and delivery vary widely, leading to inconsistencies in outcomes across studies and settings [13]. Most existing PFA programmes are structured around acute, event-based crises and use formats such as condensed workshops, short online modules, or basic informational leaflets. These typically emphasize rapid stabilization, triage of immediate needs, and short-term emotional support. However, such models are not tailored for environments like care homes, where stressors are chronic, relational, and cumulative rather than singular acute incidents. Many original PFA materials also assume access to supervisory structures, team debriefing opportunities, or organisational emergency protocols that do not consistently exist in care homes. This underscores the need to adapt PFA for specific organisational contexts, particularly for the complex and emotionally demanding environment of care homes.

Care homes are complex systems with multi-agency interactions involving GPs, hospitals, and social care services, as well as variability in resident profiles, staffing models, and organisational cultures [18,19]. As Coles et al. [20] note, interventions cannot simply be “lifted and shifted” between settings; they must be adapted to align with the specific needs, culture, and operational realities of the new environment. Furthermore, PFA sits at the level of an individual-level skills training intervention and does not replace organisational responsibilities for staff wellbeing; therefore, adaptation must clarify the intended purpose, boundaries, and mechanisms of action relevant to care homes.

Given the critical need for psychological support for care home staff, this study aimed to adapt a PFA training intervention through co-production, integrating stakeholder expertise and user feedback throughout. In this study, “co-production” refers to a structured process in which care home staff, managers, educators, mental health professionals, and researchers collaboratively contributed to decisions about training content, delivery format, language, examples, and accessibility. Consensus was reached through iterative discussion in stakeholder workshops, with decisions documented and fed back into subsequent iterations. Outputs considered part of “the intervention” included guiding principles, a logic model, curriculum content, delivery recommendations, and example scenarios. Success criteria for the development phase focused on acceptability, usability, and contextual relevance rather than effectiveness.

The goals were to contribute to a tailored adapted PFA intervention specifically for care homes to generate practical insights into its feasibility, usability, and potential effectiveness within the care home context. By co-producing a tailored PFA intervention, this study seeks to strengthen staff wellbeing, retention, and the overall quality of care provided to residents.

### Aim

The overall aim of this study is to co-produce an adapted PFA intervention for care home staff by integrating user perspectives and drawing on theory and evidence.

## 2. Methods

### 2.1. Overview of the Intervention Development Process

A person-based approach (PBA) was adopted to guide the adaptation of Psychological First Aid (PFA) training for care home settings. The PBA emphasizes iterative engagement with end-users to ensure interventions are acceptable, feasible, and aligned with their needs, values, and preferences [21]. This approach is particularly suited to complex interventions where contextual tailoring is critical for real-world effectiveness and sustainability.

The PBA in this study involved:

1. Understanding the target population and context (January–March 2023) through interviews and early stakeholder consultations.
2. Engagement and co-production with stakeholders across six monthly workshops (February–September 2023), each lasting ~90 min.
3. Development of guiding principles and a logic model (March–June 2023) using iterative stakeholder feedback and theoretical mapping.
4. Refinement of draft intervention content through qualitative feedback from think-aloud interviews (July–September 2023).
5. Preparation for subsequent feasibility testing by finalizing content and documenting implementation considerations

This study was structured into two distinct but interlinked phases: a planning and development phase (Phase 1) and an optimization phase (Phase 2). This design aligns with Medical Research Council (MRC) guidance on developing complex interventions, emphasizing iterative development, theory integration, and stakeholder involvement prior to feasibility testing [22].

### 2.2. Study Design

Phase 1 focused on generating a comprehensive understanding of care home staff needs and contextual challenges to inform the development of the intervention’s guiding principles, logic model, and content. Phase 2 employed think-aloud interviews to optimise usability and contextual relevance of the adapted materials for the care home context. These materials included newly created examples, revised language, and early versions of quizzes, visual summaries, and printable handouts. Stakeholder workshops ran throughout both phases as a distinct mechanism for ongoing co-production, ensuring decisions were informed by diverse expertise and lived experience.

#### 2.2.1. Phase 1: Planning and Development Phase

The purpose of Phase 1 was to identify how PFA training could be adapted to fit the care home environment. Activities included:

- Monthly stakeholder workshops: reviewing and sense-check interview findings, interpreting emerging qualitative themes, and co-developing the intervention framework. They followed explicit governance procedures:
  – decisions were made through consensus;
  – disagreements were recorded and revisited;
  – conflicts of interest were declared at the outset;
  – minutes were taken and used as a formal change log.
- Semi-structured interviews: exploring staff experiences, perceived training needs, and attitudes toward PFA. Sample questions included:
  – “Can you describe a situation at work that you experienced as emotionally demanding?”
  – “What type of support (formal or informal) do you usually receive after a difficult incident?”
  – “What aspects of PFA do you think might be relevant or not relevant in your setting?”
- Literature reviews: combining scoping and narrative approaches to examine the evidence base for PFA interventions and their relevance to care home contexts. In addition to the reviews by Schoultz et al. [13] and Hermosilla [23], we conducted further searches in MEDLINE, CINAHL, PsycINFO, and Google Scholar (January–February 2023). Search terms included “Psychological First Aid,” “care home,” “long-term care,” “staff wellbeing,” “trauma-informed support,” and “crisis response.” Inclusion criteria focused on PFA training studies, psychological support interventions in long-term care, and trauma-informed approaches. Outputs directly informed the guiding principles and adaptation decisions.

Phase 1 produced guiding principles, a logic model outlining hypothesised mechanisms of change, and draft training content.

#### 2.2.2. Phase 2: Optimization

Phase 2 aimed to refine and optimize the intervention through end-user testing and feedback. We utilized think-aloud interviews, an established qualitative method that captures participants’ cognitive and emotional responses as they interact with materials in real-time [24]. During the sessions, participants were asked to read material adapted from the World Health Organization’s PFA framework [7] and contextualized scenarios and headings informed by publicly available Future Learn materials [8]. Eight participants contributed to phase 1 and twelve to phase 2.

Participants verbalized their thoughts, reactions, and suggestions as they progressed through the material, allowing us to capture their real-time reflections on clarity, relevance, and usability. This approach allowed us to identify usability and comprehension challenges, understand emotional responses to the training content, and gather concrete suggestions for improving delivery, tone, and relevance. Interviews were audio-recorded and transcribed verbatim, and field notes were taken to document observations.

Interviews were audio-recorded and transcribed verbatim; field notes were taken to document contextual observations. Insights from these interviews were organized in a table of changes-[ToC] (see Supplementary Materials), systematically recording issues, participant suggestions, and corresponding modifications. Each change in the ToC linked to a specific quote, design decision, and implementation status, these adaptations were then validated through stakeholder workshops, ensuring co-produced decisions were made collectively and aligned with the guiding principles (Table 1).

### 2.3. Stakeholder Workshops

The stakeholder workshops were distinct from the participant interviews and served as an ongoing co-production mechanism to shape the intervention development process. The stakeholders met monthly, reviewing literature summaries, qualitative findings and proposed intervention materials. They provided input into all design decisions, including the refinement of interview guides and recruitment materials, the development of the logic model and guiding principles, and the review of proposed PFA content and delivery adaptations. Stakeholders did not analyze data; instead, they interpreted findings and advised adaptation decisions. Decision authority was shared: researchers led methodological decisions; stakeholders led content relevance and usability decisions. All disagreements were documented and resolved through facilitated consensus.

The stakeholder group was intentionally diverse, comprising care home educators, mental health professionals, researchers, public and patient involvement (PPI) representatives, healthcare students, and practising care home staff. This diversity of expertise ensured multi-perspective validation of decisions and enhanced the ecological validity of the final intervention.

### 2.4. Participants, Recruitment and Data Collection

Eligible participants were healthcare workers (HCWs) including carers, senior carers, nurses, and managers, employed within non-NHS care homes in the UK. Participation did not require prior exposure to Psychological First Aid (PFA) training.

Sampling was guided by stakeholder input; we employed purposive, maximum variation sampling to capture a wide range of experiences and perspectives across age, gender, role, ethnicity, and length of service. 112 individuals clicked the study link; 41 expressed interest; 22 met eligibility criteria; 18 participated (12 Phase 1; 8 Phase 2; some overlap). Recruitment used multi-channel dissemination, including professional networks such as Care Home Network, Care Home Nurses Network, Queen’s Nursing Institute Network, Local Authority Database, Care Nursing Network, nursing home-related charities, NHS Clinical Commissioning Groups (CCG), student nurses, university networks, training providers, ENRICH Scotland, and social media platforms (Twitter/X, Facebook, LinkedIn, WhatsApp). This multi-pronged strategy enhanced reach and diversity and was consistent with methods used in previous care home studies [13]. Most participants responded through Facebook and the Care Home Nurses Network.

Ethics: Ethical approval was obtained from Northumbria University Ethics Panel on 25 January 2023 (PN2471). Written informed consent was obtained from all participants and stakeholders before participating.

### 2.5. Data Collection

Semi-structured and think-aloud interviews were conducted individually and lasted up to 60 min. The stakeholder workshops, held monthly throughout the study period, lasted approximately 90 min. To promote inclusivity and accommodate varying levels of digital access and confidence, interviews and workshops were offered via Microsoft Teams, Zoom, or telephone. All workshops were audio-recorded using Microsoft Teams’ built-in recording function and logged accordingly. As recognition for their time and contribution, all participants received a £25 voucher. We acknowledge that incentives may increase participation but could also influence positive feedback.

### 2.6. Data Analysis

Data analysis followed a dynamic, iterative process consistent with thematic analysis [25] and principles of the Person-Based Approach (PBA) [26]. Transcripts from interviews and workshops, alongside field notes and co-production logs, were read repeatedly to support familiarization and reflexivity. Initial inductive coding captured participants’ experiences and perspectives, which were then organized into themes through a combined inductive–deductive approach informed by relevant theory and stakeholder insight. We used Microsoft Excell and Taguette (https://www.taguette.org/) for data management.

A framework approach was used to integrate data from stakeholder workshops, semi-structured interviews, and think-aloud sessions, ensuring transparency in how each source contributed to the development of guiding principles and the logic model. Themes were mapped onto key components of the intervention (context, inputs, activities, outputs, mechanisms of action, outcomes, and impacts), while parallel analysis identified the values and behavioral considerations informing the guiding principles.

Think-aloud data underwent sentiment analysis to capture positive, negative, and neutral responses to the training content. These insights were combined with thematic findings in a Table of Changes (ToC) (see Supplementary Materials), providing a clear audit trail of all adaptations.

To enhance dependability and rigor, 30% of transcripts were double coded by a second researcher (AK and MS), with discrepancies resolved through discussion. Throughout analysis, stakeholder workshops served as iterative feedback loops, supporting interpretation, confirming emerging insights, and ensuring that adaptations reflected end-user priorities. This co-produced analytical process provided methodological triangulation and strengthened the credibility, transparency, and transferability of findings.

## 3. Results

The findings are presented in two phases, reflecting the iterative and person-based nature of the study.

### 3.1. Phase 1: Planning and Development

Phase 1 focused on understanding care home staff experiences, training needs, and contextual factors to inform the initial design and theoretical foundations of the adapted PFA intervention. Data sources included stakeholder workshops, semi-structured interviews, and the review and interpretation of existing evidence.

A total of 18 participants and 7 stakeholders contributed to this phase. As shown in Table 2, the majority of participants were female, most were based in England, and they represented a range of ethnic backgrounds, job roles (carers, senior carers, nurses, managers), and levels of experience-from less than 5 years to over 20 years. This diversity ensured that the emerging themes reflected perspectives across different roles, levels of responsibility, and stages of professional development within care home settings.

#### 3.1.1. Themes from Semi-Structured Interviews

Thematic analysis generated five key themes representing the experiences, challenges, and needs of care home workers (Table 3). A full set of illustrative quotes is provided in the Supplementary Materials.

Role-based differences were evident: carers emphasized emotional burden and practical support needs; managers emphasized system pressures and staff shortages; nurses focused on clinical decision-making during distressing events. Ethnic minority staff more frequently reported stigma around mental health disclosure. These insights informed adaptations to ensure inclusivity, psychological safety, and relevance to multiple roles.

#### 3.1.2. Guiding Principles

Drawing on Phase 1 themes, stakeholder feedback, and prior evidence, we developed a set of guiding principles that articulated both design objectives (“what needs to be achieved”) and key features (“how to achieve it”). These ensured that intervention development remained aligned with user priorities and theoretical evidence throughout the process.

Each guiding principle was linked to a concrete design feature or adaptation (e.g., inclusion of brief reflective exercises, voice-over options for accessibility, or scenario-based modules). These principles represent a key output of Phase 1 and a methodological product of the PBA process, contributing to the originality and transferability of this work. These mappings are detailed in the new Appendix A Table A1.

The original WHO PFA materials served as the conceptual foundation; the adapted version incorporates care-home contextual needs identified during Phase 1 and usability feedback refined during Phase 2.

#### 3.1.3. Logic Model

**Figure 1:**
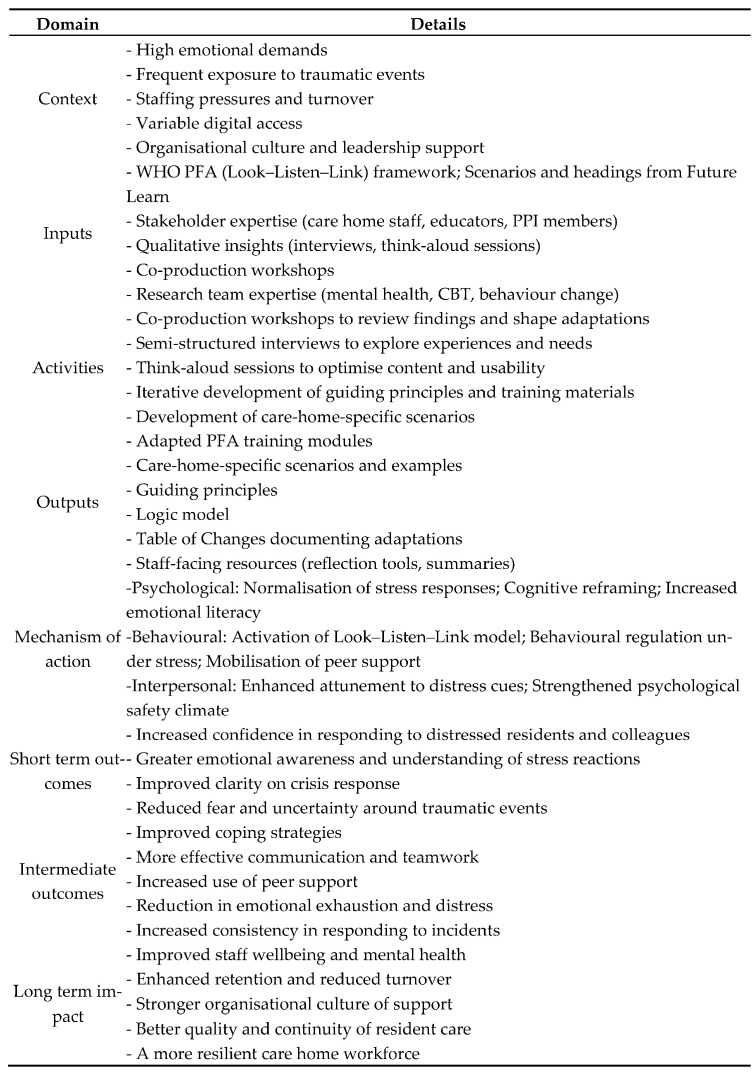
Logic model.

A logic model (Figure 1) was developed to articulate how the adapted PFA intervention is hypothesized to work. Rather than restating project activities, the model identifies the key psychological and behavioral mechanisms through which training is expected to influence staff wellbeing. These mechanisms include the normalization of stress responses, activation of the PFA ‘Look–Listen–Link’ process, cognitive reframing, development of self-efficacy, improved behavioral regulation under stress, increased interpersonal attunement, and mobilization of peer support. These processes are theorized to lead to short-term improvements in emotional literacy and confidence, intermediate outcomes such as improved coping, communication and reduced distress, and longer-term impacts on staff wellbeing, retention, and quality of resident care.

The logic model functioned as a dynamic tool, updated iteratively as Phase 2 findings emerged, ensuring continuous alignment between user insight, theory, and intervention design. This integration of mechanisms of action with contextual realities represents a conceptual contribution to the adaptation of trauma-informed interventions for complex care environments.

### 3.2. Phase 2: Optimisation and Refinement

Phase 2 employed think-aloud interviews with 12 participants to evaluate the acceptability, clarity, and usability of the unadapted PFA training materials. Data generated 129 distinct codes (39 positive, 30 negative, and 60 neutral). A sample of these findings is available in Table 4.

#### 3.2.1. Key Findings

1. Training Effectiveness and Engagement:

Participants valued the structured nature of PFA and its potential to guide responses during emergencies. Realistic scenarios and clear instructions enhanced perceived usefulness, while repetitive or overly lengthy sections reduced engagement.

2. Information Clarity and Ease of Understanding:

Content was generally seen as clear and comprehensible. Participants felt more confident and less fearful in their roles after engaging with the material, suggesting potential for empowerment and improved communication.

3. Format and Delivery Challenges:

Repetitive text, limited interactivity, and long duration were identified as barriers. Participants recommended shorter, modular delivery and more engaging learning formats such as videos or case studies.

4. Technological Accessibility:

Digital literacy and access issues were raised, highlighting the need for multiple modes of delivery (e.g., mobile, printed, or blended learning options).

5. Staff Engagement and Perceptions:

Some staff expressed reluctance to discuss mental health openly due to perceived stigma or fear of judgement, underlining the importance of psychological safety in training delivery.

6. Recommendations for Improvement:

Participants suggested including care-home-specific case studies, scheduling training during work hours, and incorporating interactive exercises (e.g., group discussions, role-play, or reflection prompts) to promote engagement.

#### 3.2.2. Integration of Findings

Findings from the think aloud activity were discussed during stakeholder workshops, and adaptations were prioritised according to relevance, feasibility, and alignment with the guiding principles. The final co-produced version of the adapted PFA training incorporated:

- Bite-sized interactive modules for flexible learning,
- Care-home-specific scenarios and examples,
- Blended delivery options (online and printable), and
- Built-in reflection and peer support prompts to sustain engagement.

These refinements enhanced the contextual fit, accessibility, and acceptability of the intervention, laying the foundation for feasibility and acceptability testing in future research. See Table S1.

## 4. Discussion

### 4.1. Summary of Findings

This study aimed to co-produce an adapted Psychological First Aid (PFA) training intervention specifically for care home staff, enhancing its relevance, acceptability, and feasibility within the unique context of care homes. Using a person-based approach (PBA), we developed guiding principles, identified essential content modifications, and produced an adapted version of PFA that reflects staff experiences, preferences, and organisational constraints. Importantly, the study focused on adaptation and optimisation rather than outcome evaluation, and therefore any references to potential benefits should be interpreted as perceived usefulness rather than demonstrated impact.

### 4.2. Interpretation of Key Findings

A central finding across both phases was that the original PFA materials—while theoretically robust, did not sufficiently align with the daily realities of care homes. Staff described the need for training that reflected the types of traumatic events they routinely encounter, such as sudden resident deterioration, family distress, or escalating behaviors in dementia. This insight reinforced the importance of contextualized case studies, which were incorporated into the adapted intervention to capture the emotional and relational demands of care home practice. This goes beyond reaffirming the need for trauma-informed support established in earlier literature [3,27] by demonstrating how adaptation can operationalize these principles in a complex care setting.

Another major finding concerned training format and delivery. Staff consistently reported that traditional e-learning or lengthy online modules were difficult to complete due to workload pressures, staffing shortages, and unpredictable shift patterns. The adapted PFA resource therefore adopted bite-sized, interactive modules, designed to be accessible, concise, and easy to pause and resume. This adaptation is supported by evidence demonstrating that short, modular digital learning [28] enhances engagement and knowledge retention in healthcare settings [29]. Recent work in care home contexts [30] similarly found that long training sessions were impractical and contributed to staff dropout—highlighting the importance of flexible, time-efficient learning formats. Participants also emphasized barriers related to digital literacy and variable access to online platforms. To address this, the adapted intervention includes blended delivery options, with printable materials available alongside online content. This approach reflects evidence that hybrid delivery supports inclusivity and reduces inequalities in digital access across the care home workforce.

A further refinement involved incorporating reflective prompts and optional peer-support components. Staff expressed a desire for support that normalizes emotional reactions and encourages communication without requiring personal disclosure. Including structured reflection therefore enhances both acceptability and psychological safety, consistent with trauma-informed principles and workplace mental health research [31]. However, such acceptability was based on staff perceptions during development; no conclusions can be drawn about actual emotional or behavioral change until the intervention is evaluated.

Taken together, these adaptations demonstrate that acceptability of PFA in care homes hinges not only on content relevance but also on delivery format, emotional tone, and recognition of local work constraints. Findings reflect staff perceptions of usefulness and relevance rather than tested impacts on confidence, communication, or wellbeing. 

This study illustrates how co-production can surface such nuances and ensure that an intervention is practically implementable within a demanding care environment.

### 4.3. Theoretical and Methodological Contributions

This research provides a transparent example of adapting an evidence-based psychological intervention for a new context using the person-based approach. By grounding modifications in trauma-informed care principles and behavioral mechanisms (such as enhanced emotional literacy, improved self-efficacy, and strengthened interpersonal attunement), we developed a logic model illustrating how the adapted PFA intervention is expected to lead to improved coping, communication, and wellbeing. The cognitive reframing and behavioural regulation were theory-informed rather than directly derived from data and should therefore be considered proposed rather than empirically demonstrated mechanisms. 

Unlike earlier PFA research, which has predominantly focused on healthcare or humanitarian settings [12,23], this study shows how PFA can be shaped to address the relational, emotional, and organisational characteristics of care homes. The co-produced guiding principles and logic model provide a theoretically grounded foundation for future feasibility and evaluation studies, in line with the MRC Framework for Developing and Evaluating Complex Interventions [22].

This work therefore extends the existing literature by positioning PFA not as a generic crisis-support tool but as a context-specific, culturally attuned resource for care home practice. The methodological integration of sentiment analysis, iterative co-production, and PBA principles represents an innovative approach to intervention adaptation, with potential transferability to other high-stress care environments internationally. We acknowledge that sentiment analysis may simplify nuanced qualitative data; its purpose here was limited to assessing emotional reactions during usability testing rather than providing in-depth interpretive insights. Co-production added contextual and practical rigour, but it also introduces potential ambiguity regarding the boundary between data and design inputs, an issue we have now made explicit.

### 4.4. Practical Implications

The adapted PFA resource has the potential to enhance care home staff’s confidence, communication, and preparedness during critical incidents, while supporting a more open culture around emotional wellbeing. However, these perceived benefits should not be interpreted as demonstrated outcomes. The intervention’s actual impact on confidence, communication, or wellbeing requires empirical testing. 

Implementation, however, will require organisational commitment, including allocating protected time for completion, recognizing the emotional demands of care work, and embedding PFA within existing supervision or wellbeing structures. Without these enabling conditions, even well-designed training may struggle to achieve uptake.

### 4.5. Comparison and Broader Implications

Our findings align with previous research on PFA in healthcare and humanitarian contexts, which emphasizes the importance of psychological safety and structured support during crises [32,33]. However, this study extends the evidence base by demonstrating how PFA can be adapted for the relational and organisational complexities of care homes through co-production and person-based principles. The integration of blended delivery formats and accessibility features addresses digital inequalities, promoting inclusivity across a diverse workforce [34]. These adaptations have international relevance, offering a transferable model for tailoring trauma-informed interventions to other high-stress care environments globally [34,35]. Furthermore, this work supports WHO recommendations and aligns with policy priorities for workforce resilience and mental health in post-pandemic recovery strategies. We also acknowledge ongoing debates about PFA effectiveness and fidelity across settings. These debates highlight the importance of ensuring fidelity to core principles (e.g., “Look–Listen–Link”) while adapting peripheral components. This study contributes by documenting exactly how core elements were preserved and how contextual adaptations were justified.

### 4.6. Limitations

This study has several limitations. First, some degree of recruitment bias is possible, as participants may have had a greater interest in wellbeing or training. Second, data relied on self-report through interviews and think-aloud activities, which may be influenced by recall or social desirability bias. Third, the study was conducted in UK non-NHS care homes, which may limit transferability to other systems or international contexts. Finally, researcher interpretation is inherent to qualitative research; however, the use of triangulation, reflexive discussions, and stakeholder validation strengthened credibility. In addition, several methodological considerations should be acknowledged. Co-production processes may introduce subtle power dynamics between researchers and participants, and although efforts were made to create an open and inclusive environment, this influence cannot be entirely ruled out. Similarly, while reflexivity was embedded in team discussions, more explicit reporting of researcher positionality could further enhance transparency. The study also involved both empirical data collection and collaborative design work, and although these were treated as distinct processes, the boundaries between data informing findings and feedback informing adaptations may occasionally overlap. Finally, sentiment analysis, used to capture immediate emotional reactions during the optimization phase, provides a useful but simplified categorization of participant responses and should be interpreted alongside, rather than in place of, the richer thematic findings. These additional considerations reflect the complexity of adapting psychological interventions in real-world settings and highlight areas for refinement in future research.

### 4.7. Future Research Directions

Future work should assess the feasibility, usability, and acceptability of the adapted PFA training within real-world care home environments. Before moving to trials, key feasibility questions include: minimum organisational conditions (e.g., protected time), staff completion rates by shift/role, digital access needs, training fidelity, and implementation costs. Pilot testing should explore delivery modes, completion rates, and barriers to implementation. Subsequently, a cluster randomized controlled trial could examine the intervention’s impact on staff wellbeing, burnout, and communication, as well as resident outcomes. However, such trials should only proceed once feasibility criteria are clearly met. Process evaluations will be essential to understand mechanisms of action and contextual influences on implementation. Research should also consider longer-term sustainability and strategies for embedding PFA within organisational policy and ongoing professional development. Future research should focus on feasibility testing, process evaluation, and effectiveness trials to establish impact and sustainability. This work provides a transferable model for adapting psychological interventions to other high-stress care environments internationally, advancing the evidence base for trauma-informed workforce support.

## 5. Conclusions

This study demonstrates the feasibility of adapting an evidence-based Psychological First Aid (PFA) intervention for care home staff using a person-based, co-production approach. It does not provide evidence of feasibility of implementation or effectiveness in practice, and we therefore clarify that our conclusions relate only to the adaptation process. By integrating stakeholder insights, qualitative feedback, and theoretical principles, we developed guiding principles, a logic model, and an adapted training resource tailored to the emotional, organisational, and practical realities of care homes. The resulting intervention addresses critical gaps in workforce resilience and trauma-informed support by combining context-specific content with flexible, accessible delivery formats.

These findings have important implications for policy and practice. Implementing PFA in care homes could strengthen staff wellbeing, confidence, and communication during critical incidents, contributing to improved care quality and retention. Such outcomes remain hypothetical until evaluated. However, successful adoption will require organisational commitment, protected time for training, and integration within existing wellbeing structures.

## Figures and Tables

**Table 1:** Guiding Principles for the Adapted Psychological First Aid (PFA) Training Intervention.

1.	Address individual staff needs, perceptions, and preferences regarding psychological support and coping.
2.	Reflect the diversity of traumatic events encountered in care homes and tailor materials to this heterogeneity.
3.	Balance structure with flexibility to accommodate varying roles and responsibilities.
4.	Deliver training through short, modular, and accessible online sessions that fit shift patterns and digital literacy levels.
5.	Incorporate realistic care-home-specific examples and scenarios to enhance relevance and engagement.
6.	Promote reflective practice and proactive self-care to strengthen emotional resilience.
7.	Ensure inclusivity, accessibility, and psychological safety within all training materials.
8.	Integrate with existing care home systems and policies to maximize feasibility and sustainability.
9.	Encourage peer learning and ongoing skill reinforcement through discussion and support networks.
10.	Embed the core PFA principles of “Look, Listen, Link” while adapting language and application to the care home context

**Table 2:** Study participants and stakeholders.

Characteristics	Participants		Stakeholders	
	Female	Male	Female	Male
Country Total N = 18			Total N = 7	
England	12	2	5	2
Scotland	3	1	0	0
Age				
16–24	0	1	0	0
25–44	9	1	2	1
45–64	5	1	3	1
65+	1	0	0	0
Ethnicity				
White	11	1	3	0
Asian	3	1	1	1
Black/African/Caribbean	4	1	1	1
Other	2	0	0	0
Qualification				
NVQ 1 or 2	1	0	1	0
NVQ 3	1	1	1	1
Diploma	1	0	0	0
Degree	9	0	2	0
Postgraduate or above	2	2	1	1
Other	1	0	0	0
Job Title				
Carer	4	2	0	0
Senior Carer	1	0	0	0
Nurse	2	0	0	0
Manager	7	1	0	0
Other (students, retired, educators and PPI)	1	0	5	2
Years of experience in the job				
Less than 5	1	1	1	1
6–10	3	0	1	0
11–15	2	0	2	0
16–20	2	1	0	1
20+	7	1	1	0

**Table 3:** Themes capturing the experiences and challenges.

Traumatic Events in Care Homes:	Staff commonly described emotionally distressing events such as resident deaths, sudden health deterioration, and conflict with relatives. These incidents were experienced as “constant occurrences” that contributed to emotional exhaustion.
Support and Coping Mechanisms:	Participants reported limited organisational and peer support following traumatic events. Coping strategies were often individual and informal, and perceived mental-health-related stigma discouraged open discussion.
Measurement of Training Effectiveness:	Staff expressed a desire for clear indicators of success (e.g., pre- and post-training surveys, monitoring of sickness absence) to evidence training impact.
Transferability of Skills:	Participants valued training that built transferable communication and coping skills, applicable both inside and outside care home settings.
Motivators for Development:	Key motivators included personal fulfilment, professional growth, and a desire to improve care for residents.

**Table 4:** Sample Codes from Think-Aloud Interviews.

Theme	Positive Sentiments	Negative/Neutral Sentiments	Adaptation Implemented
Training structure	“Provides clear steps and calm procedures.”	“Too long and repetitive.”	Divided into shorter modules with clearer sectioning.
Relevance	“Scenarios felt realistic and relatable.”	“Some examples not care-home-specific.”	Replaced examples with care-home scenarios (e.g., resident death, family conflict).
Accessibility	“Easy to understand.”	“Not all staff comfortable with online tools.”	Added printable materials and offline options.
Engagement	“Liked the reflective parts.”	“Too much text; needs more interaction.”	Added quizzes, short videos, and reflection prompts.
Emotional safety	“Makes me think about my own wellbeing.”	“Some staff may not feel safe sharing feelings.”	Embedded guidance on confidentiality and self-care.

## Data Availability

De-identified qualitative data (interview transcripts and Table of Changes) are available in the Supplementary Materials. Additional data may be provided upon reasonable request to the corresponding author.

## Supplementary Materials

The following supporting information can be downloaded at: https://www.mdpi.com/article/10.3390/ijerph23040431/s1, Table S1: Table of Integrated Findings; File S1: De-identified qualitative data.

## Appendix A

**Table A1:** Comparison of original WHO Psychological First Aid (PFA) materials and the adapted care-home PFA training intervention.

Training Element	Original WHO PFA Materials	Adapted PFA for Care-Home Context
Format	Text-heavy manual; primarily narrative guidance	Short, modular sessions with clear sectioning; designed for 10–15-min learning blocks
Delivery Mode	Self-guided reading; facilitator-led in emergencies	Blended delivery (online modules + printable quick-reference materials)
Examples & Scenarios	Disaster, emergency, and humanitarian crisis scenarios	Care-home-specific scenarios (e.g., resident death, family conflict, sudden deterioration)
Learning Activities	Minimal structured activities	Interactive quizzes, reflection prompts, micro-case studies, and short videos
Language Style	Generic humanitarian phrasing	Contextualized, simplified, care-home-specific language and terminology
Emotional Safety Content	Basic advice on self-care	Expanded self-care guidance, confidentiality reminders, supportive reflection exercises
Accessibility Features	Standard text formats	Printable sheets, mobile-friendly formats, low-literacy adjustments, optional audio narration
Role Relevance	Not tailored for specific workforce groups	Adapted pathways for carers, senior carers, nurses, and managers
