# Supplementary material for: Adapting and Co-Producing a Psychological First Aid Intervention for Care Home Staff: A Person-Based Approach to Enhance Workforce Resilience
Source: Int J Environ Res Public Health. 2026 Mar 30;23(4):431. doi: 10.3390/ijerph23040431 (PMC13116527; doi:10.3390/ijerph23040431)
Supplement: Supplementary file 1 [file ijerph-23-00431-s001.zip › Table S1.pdf]

**Table S1.** Table of Integrated Findings.

| Intervention Domain | Original Materials (WHO + FutureLearn elements) | Identified Issues (Negative/Neutral Sentiments) | Positive Sentiments | Final Adaptation (Co-produced Version) | Example Change |
|---|---|---|---|---|---|
| Training Structure | Long, text-heavy modules; limited signposting of PFA steps | Perceived as *too long*, repetitive; unclear structure initially | Clear steps appreciated | Re-structured into short, bite-sized modules with clear sectioning and upfront explanation of Prepare–Look–Listen–Link | Training divided into 10–15 min segments |
| Relevance of Content | Generic emergency scenarios; some COVID-only examples; references to children/LD | Not care-home-specific; irrelevant examples reduced engagement | Scenarios appreciated when relatable | Replaced generic scenarios with care-home-specific ones addressing resident deterioration, sudden death, distressed relatives, and dementia-related escalation | *Scenario added*: Rapid overnight deterioration + distressed daughter |
| Language & Clarity | Technical terms (hypervigilance, psychosocial, altruism); some confusing phrasing | "Pitched too high"; confusing quiz wording; unclear definition of PFA | Clear parts were well-received | Simplified terminology, reworded questions, clearer introduction explaining what PFA is and why it is relevant in care homes | Terms replaced with plain English; quizzes rewritten |
| Knowledge Checks | Simple, broad quizzes | Not challenging or specific enough | Staff liked having quizzes | Added more varied, care-home-specific knowledge checks | Quiz includes recognising resident distress cues |
| Scenarios & Application | Limited coping examples; some black-and-white portrayals of stress responses | Lack of practical coping strategies; too simplistic | Realistic examples valued | Added reflective exercises, coping strategy examples, and more nuanced stress-response descriptions | New section on staff emotional reactions and normalisation |
| Delivery Format | Online, video-led; some unengaging videos | Digital confidence varies; some videos boring; online format can limit discussion | Videos liked when short and focused | Blended delivery (online + printable options); unengaging videos removed; content shortened | Printable workbook added |
| Engagement & Interactivity | Primarily text-based learning | Too much text; insufficient interaction | Reflective parts appreciated | Added quizzes, micro-activities, and short reflective prompts to increase active engagement | "Pause and reflect" boxes incorporated |
| Emotional Safety & Support | Limited focus on staff emotional response | Staff may feel unsafe discussing distress; stigma concerns | Positive value placed on wellbeing focus | Added guidance on confidentiality, normalisation of emotional responses, and optional peer-support prompts | Reflection prompts emphasise voluntary participation |
| Relevance Beyond COVID-19 | Strong COVID-19 emphasis in FutureLearn materials | Felt outdated or irrelevant | COVID reflection still valuable | COVID content reframed as reflective learning, not core content | Short reflective box rather than whole module |
